# Cognitive Enhancement Therapy vs social skills training in schizophrenia: a cluster randomized comparative effectiveness evaluation

**DOI:** 10.1186/s12888-022-04149-x

**Published:** 2022-09-01

**Authors:** Russell K. Schutt, Haiyi Xie, Kim T. Mueser, Matthew A. Killam, Jonathan Delman, Shaun M. Eack, Raquelle Mesholam-Gately, Sarah I. Pratt, Luis Sandoval, Meghan M. Santos, Laura R. Golden, Matcheri S. Keshavan

**Affiliations:** 1 Beth Israel Deaconess Medical Center, Harvard Medical School, Boston, USA; 2 University of Massachusetts Boston, Boston, USA; 3 Dartmouth College, Hanover, USA; 4 Boston University, Boston, USA; 5 University of Massachusetts Medical School, Worcester, USA; 6 University of Pittsburgh, Pittsburgh, USA; 7 Geisel School of Medicine at Dartmouth, Hanover, USA

**Keywords:** Schizophrenia, Social skills training, Cognitive enhancement therapy, Cognitive remediation, Community functioning, Cluster randomized controlled trial, Psychosocial rehabilitation, Neurocognition, Social cognition

## Abstract

**Background:**

Schizophrenia and related disorders are highly disabling and create substantial burdens for families, communities, and health care systems. Although pharmacological treatments can often lessen the psychotic symptoms that are a hallmark of schizophrenia, they do not lessen the social and cognitive deficits that create the greatest impediments to community engagement and functional recovery. This study builds on prior research on psychosocial rehabilitation by comparing the effectiveness of two treatments demonstrated as efficacious in improving social and community functioning, Cognitive Enhancement Therapy (CET) and a version of Social Skills Training (HOPES/SST).

**Methods:**

The study uses a randomized cluster design in which a pair of clinicians at community- and hospital-based mental service centers deliver either CET or HOPES to at least one group of 6-8 eligible clients for 12 months. Clinicians are trained and then supervised weekly, with ongoing process measurement of treatment fidelity, attendance, satisfaction, and retention, and use of other services. Measures administered at baseline and at 6 and 12 months while in treatment, and then at 18 and 24 months after treatment include social adjustment, quality of life, social skills, positive and negative symptoms, and neuro- and social cognition. We hypothesize that CET will be associated with greater improvements than SST in both the primary outcome of community functioning and the secondary outcomes of neuro- and social cognition and social skills. Secondarily, we hypothesize that more cognitive impairment at baseline and younger age will predict more benefit from CET compared to HOPES.

**Discussion:**

Resource shortages endemic in mental health services and exacerbated by the pandemic highlight the importance of identifying the most effective approach to improving social and community functioning. We aim to improve understanding of the impact of two efficacious psychosocial treatments and to improve clinicians’ ability to refer to both treatments the individuals who are most likely to benefit from them. We expect the result to be programmatic improvements that improve the magnitude and durability of gains in community functioning.

**Trial registration:**

ClinicalTrial.gov NCT04321759, registered March 25, 2020.

## Background

Schizophrenia (SZ) and related disorders occur in between .4-1% of the population across the world—about 2 million individuals in the United States, with 100,000 new cases diagnosed each year. They are highly disabling, create substantial burdens for their families and communities [1–3], and cost the US more than $100 billion annually [4, 5]. Although psychotic symptoms are the most florid manifestations of SZ and related psychotic disorders [6], associated impairments in social and cognitive functioning are the most disabling features of this diagnosis [7, 8]. While pharmacological treatments can lessen positive symptoms for many individuals [9–11], they do not improve social or cognitive functioning [7, 12–18]. As many as two-thirds of people with SZ have social skills impairments [19, 20], over 90% are impaired in one cognitive domain, and 75% are impaired in at least two domains [21–24]. Impaired cognitive and social cognitive functioning are also rate-limiting factors in response to psychosocial treatment and constrain prospects for community engagement and functional recovery [19, 20, 23, 25–30].

An array of psychosocial programs to improve social skills and cognitive functioning have been developed in recent decades. Social skills training (SST) is a common approach to improving social functioning, most often provided in a group format, aimed at teaching more effective interpersonal skills. Skills are taught based on the systematic application of social learning theory, and include the following steps: breaking complex skills down into smaller steps or components, demonstrating (modeling) the skill for participants in a role play, engaging each participant in practicing the skill in role plays with each one followed by positive feedback and suggestions for improvement, collaboratively developing home assignments for participants to practice the skills on their own, and in vivo community trips to practice the skill. Considerable evidence supports the efficacy of SST in improving social and community functioning as well as reducing negative symptoms [23, 24].

Cognitive remediation (CR) programs take a different approach to improving functioning, with a focus on enhancing cognition through practice of computer cognitive exercises, coaching in strategies designed to improve performance on the cognitive exercises, and teaching self-management (or compensatory) skills to improve cognition in day-to-day situations. Although most cognitive remediation programs have produced moderate gains in overall cognitive performance [31, 32], by themselves they have had limited lasting, positive effects on more ecologically valid abilities that shape community functioning [33]. It is when cognitive remediation programs also include strategic training and more comprehensive integration with other psychosocial interventions that they have more effects on functioning [32, 34–37]. Meta-analyses suggest that such comprehensive programs have stronger effects on community functioning than either treatment administered separately [37, 38]. However, these more comprehensive treatments have not been compared directly to traditional SST programs that do not include a CR component.

The Patient-Centered Outcomes Research Institute (PCORI) funded Project SUCCESS, Schizophrenia: Understanding and Comparing Cognitive Enhancement and Social Skills training to fill this knowledge gap and thus inform the treatment decisions of clinicians, individuals with SZ, and other stakeholders. Meta-analyses and independent randomized controlled trials (RCTs) of comprehensive CR treatment, including Cognitive Enhancement Therapy (the one we will provide) indicate moderate to large effect sizes (Cohen’s *d* between .4- 1.0, averaging .8) on cognition, social cognition, and community functioning in both early course and multi-episode patients with SZ (see Table 1) [21, 22, 32, 37, 39, 43]. By comparison, meta-analyses of RCTs testing SST treatments show small to moderate effect sizes (about .2-.5) on social skills and community functioning [23, 24, 44]. Improvements in community functioning are sustained after both treatments for at least 1-2 years [41, 42, 45–48].

**Table 1:** Effect Sizes for RCTs of CET and SST in Schizophrenia

Study	Design	Interventions	Effect sizes
Eack et al. 2009 [39]	RCT	CET vs enriched supportive therapy	.82^a^, 1.53^b^ social adjustment (SAS-II); 1.08^a^, 1.55^b^ social cognition
Wojtalik et al. 2021 [40]	RCT	CET vs enriched supportive therapy	.74^a^ role functioning
Bartels et al. 2014 [41]; Mueser et al. 2010 [42]	RCT	HOPES (SST) vs TAU	.20^a^, .29^b^,.08^c^ social functioning (SBS) .25^a^, .32^b^, .25^c^ independent living (ILSS) .44^a^, .37^b^, .26^c^ community functioning (Multnomah CAS) .54 ^a^, .53^b^, .27^c^ negative symptoms (SANS) .51 ^a^, .45^b^, .27^c^ social competence (UPSA)
Kurtz et al. 2008 [23]	Meta-analysis of RCTs	SST vs TAU	.47 negative symptoms .41 psychosocial functioning
Turner et al. 2018 [24]	Meta-analysis of RCTs	SST vs TAU	.32 (PANSS) .19 (negative symptoms) .41 (social competence)

^a^12-month effect sizes, ^b^24-month effect sizes; ^c^36-month effect sizes

## Methods

### Aims and hypotheses

**Fig. 1:**
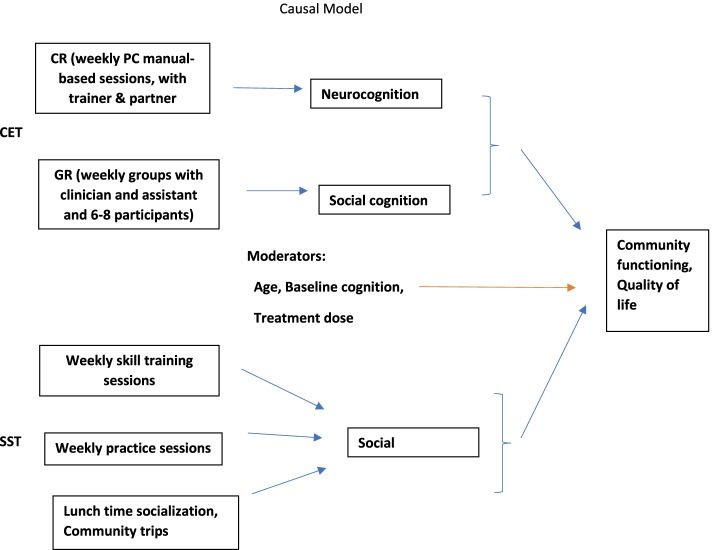
Causal Model

Our primary study aim is to test the hypothesis *that CET will be associated with greater improvements than SST* in both the primary outcome—community functioning—and the secondary outcomes of neuro- and social cognition and social skills (see Fig. 1).

Our secondary aim is to explore the differential effectiveness of the two interventions by baseline cognitive functioning and age. Because integrated treatment programs like CET were designed to reduce the moderating effects of impaired cognition on response to social skills training [20, 23], we predicted that *poorer baseline cognitive functioning* will be associated with *greater treatment response* for patients who receive CET as compared to those who receive SST (which doesn’t seek to increase cognitive functioning) [32, 37, 49–56]. Given that the younger brain retains neuroplastic reserves that may make it uniquely capable of benefiting from cognitive remediation [57–59], we also predicted that younger participants would benefit more from CET than older ones, while the same interaction with age would not occur for SST recipients [60–62]. Variation across our treatment delivery sites (approximately 24) in treatment dose may also influence the effectiveness of both interventions, with individuals attending more treatment sessions—even when it is due to challenges at the site rather than orientations of the participants—potentially benefiting more from the treatment.

### Trial design

Project SUCCESS is a comparative effectiveness trial of Cognitive Enhancement Therapy (CET), a combined treatment offering both CR and features designed to improve social functioning, with Helping Ourselves Pursue and Experience Success (HOPES), a treatment that offers only SST. The study uses a cluster-randomized design to allocate participating treatment sites to provide either CET or HOPES in groups of 6-8 eligible participants for 12 months of treatment, with post-treatment assessments of functioning conducted up to 12 months post-treatment. Most sites will offer two successive treatment groups.

### Study setting and sample

Treatment sites are approximately 24 community mental health centers and hospital outpatient clinics in Massachusetts and eight other states that offer programs for individuals with schizophrenia-spectrum disorders (final list on project website, project-success.net). These sites have been paired by geographic proximity, organizational type, and by race and ethnic proportions in the local community and then randomly assigned to the two study arms, so that the number of participants per arm is approximately equal and the distribution of sites is comparable geographically and unbiased with respect to participant and site characteristics.

Cluster randomization was used to reduce challenges created by the group-based nature of both treatments. We also had several other reasons to reject person-level randomization: increased resources required by participating sites to train two sets of clinicians to implement CET and SST, potential cross-contamination of the two interventions offered within the same setting, and slower accrual of groups in each treatment due to randomization to two different programs.

### Study interventions

*Cognitive Enhancement Therapy* is a comprehensive cognitive remediation program designed to maximize gains in social functioning by integrating computer-based training to enhance neurocognition with group-based exercises to improve social cognition [22, 43, 45, 57]. CET facilitates the development of adult social-cognitive milestones (e.g., perspective-taking, social context appraisal) by shifting thinking from reliance on effortful, serial processing to a “gistful” and spontaneous abstraction of social themes. All procedures are described in a step-by-step manual followed by CET trainers [45]. CET’s group-based exercises are delivered for 1.5 hours each week in a group of 6-8 participants led by two clinicians. During each of three modules (basic concepts, social cognition, CET applications), the group focuses on acquisition of adult social milestones in perspective-taking, social context appraisal, and other aspects of social functioning, with psychoeducational lectures on topics ranging from the neurobiology of schizophrenia to the concept of self-defeating thinking, homework assignments, and in-group exercises. CET’s computer-based training is supervised by a clinician who meets weekly for one hour with a pair of participants who make progress on an attention, memory, or problem-solving exercise in each session. The paired participants take turns practicing exercises on a computer, providing encouragement and advice to each other, as the clinician provides strategy coaching and support.

*Social Skills Training*: HOPES is a manualized SST rehabilitation program that is delivered over a 12-month period, with weekly group skills classes and monthly community practice trips and engagement of natural supports to facilitate skill use in real-world situations. HOPES classes are conducted using the principles of SST (modeling, role playing, positive and corrective feedback, in vivo assignments), some of it adapted from the Bellack et al. [63] curriculum. The curriculum is organized into eight topic areas or “modules”: Living Well in the Community, Communicating Effectively, Making and Keeping Friends, Making the Most of Leisure Time, Using Medications Effectively, Healthy Living, Intimacy and Dating, and Making the Most of a Doctor’s Visit. Each module includes 6–8 component skills, with one skill covered each week. HOPES is standardized in a manual and a participant workbook with handouts for each skill [48].

Two modifications have been made in the interventions and accompanying manuals to adapt CET and HOPES for the unique needs of this multi-site comparative effectiveness study.

1. 12-month intervention

Although the early studies of CET provided treatment for 24 or 18 months, CET has been adapted to be delivered as a 12-month intervention to enhance feasibility of delivery; it has been shown to be efficacious even when limited to 9 months (effect size of .74 for role functioning after 9 months of CET) [40]. Most SST programs are 6-9 months in length [24], but HOPES was designed as a 12-month intervention. We offer both CET and SST (HOPES) for 12 months to enhance the likelihood that participants will complete the entire program while allowing recruitment of new cohorts, and to standardize the length of treatment across groups. Evidence supports the effectiveness of both interventions by 12 months of treatment, with durability of effects at one-year follow-up for both CET [45, 46] and SST [42]—including for HOPES [41].

For CET, participants will be engaged in both the neurocognitive training and group-based social cognition training concurrently, upon enrollment. Although the CR component of CET has typically been initiated 3 months prior to the start of the group therapy sessions, we have found that sequential timing of CR exercises and group interventions is both impractical and unnecessary. Additionally, increasing evidence suggests that near-term transfer (immediate improvement in a test situation) of skills learned in cognitive remediation is likely to occur simultaneously with generalization to daily tasks and improve long-term transfer [58, 64, 65]. This has proven to be successful both in our own research programs and related implementation efforts [66]. This strategy also assures similar treatment lengths, without limiting our ability to answer the main study questions. We have developed a supplement to the CET manual describing adaptations for the 12-month intervention.

2) Replacement of dropouts in first month (partial rolling admission) for CET groups

HOPES typically uses an open enrollment format where participants can join the group at any point in the curriculum, which is taught on a revolving basis for as long as needed with the last module followed by the first module, and then remain in the program for a full year. An open enrollment format can minimize the time participants have to wait to join a new group, and is feasible in HOPES because the skills taught in the different modules are not cumulative, and thus can be learned in any order. Fully open enrollment is not feasible in CET, since some of the later modules (such as CET application to everyday life situations) require prior practice of the cognitive skills. The standard CET approach instead has been to allow replacement of dropouts in the first month of treatment, with some preliminary coaching of those who enter in weeks 2-4. We therefore have also imposed a first-month limit for enrollment to standardize this aspect of both treatments.

#### Adaptations for the COVID-19 pandemic

The COVID-19 pandemic required an abrupt and massive shift of community mental health services to telehealth modes of service delivery. Prior research indicated the potential for remote delivery of psychosocial interventions, but mostly in relation to individual therapy rather than group-based treatments [67–76]. We adapted our intervention delivery and research procedures to maintain equivalent service experiences and assessment protocols even when services and assessments must be delivered remotely. Monthly group community trips in HOPES were changed to virtual monthly meetings of each participant with an identified support person. Virtual group (and individual) sessions in both interventions and computer sessions in CET were offered on tablets or laptops. We have tested these telehealth adaptations for acceptability and feasibility in a 3-month pilot study at four service sites, two of which were assigned to CET and two to HOPES, with one in each condition delivering the intervention via telehealth and the other delivering the intervention in-person, as allowed by site policy and with adherence to site-specific public health requirements (e.g., social distancing, masking). Data on treatment enrollment, retention, participant satisfaction, and treatment fidelity indicate the feasibility and acceptability of the telehealth approach.

### Eligibility and randomization

Potential participants are eligible if the referring clinician reports a DSM-5 diagnosis of schizophrenia, schizoaffective disorder, or schizophreniform disorder that is confirmed by a Mini International Neuropsychiatric Interview (MINI) for Psychotic Disorders Studies [77] administered via video conference by the project research coordinator. Participants must also be 18-65 years of age and have an estimated premorbid IQ > 70 (determined with the Wechsler Test of Adult Reading) and have no known organic neurological disease or intellectual disability (DSM-5). Prior to assessment, on-site research assistants review the project with potential participants and invite their participation if they sign a site- and treatment-specific Informed Consent Form.

Assessments of symptom severity, social cognition, and community functioning will be conducted via video conference by trained research assistants at the coordinating site, Beth Israel Deaconess Medical Center (BIDMC), who are blind to treatment condition. Treatment fidelity will be assessed by coding at least 10 recorded group therapy sessions each year for each treatment site, with 6 sessions in the first 3 months used for feedback and training and 4 or more at random intervals after that. Site research assistants will record participant attendance and engagement during group sessions.

### Sample size and power

**Fig. 2:**
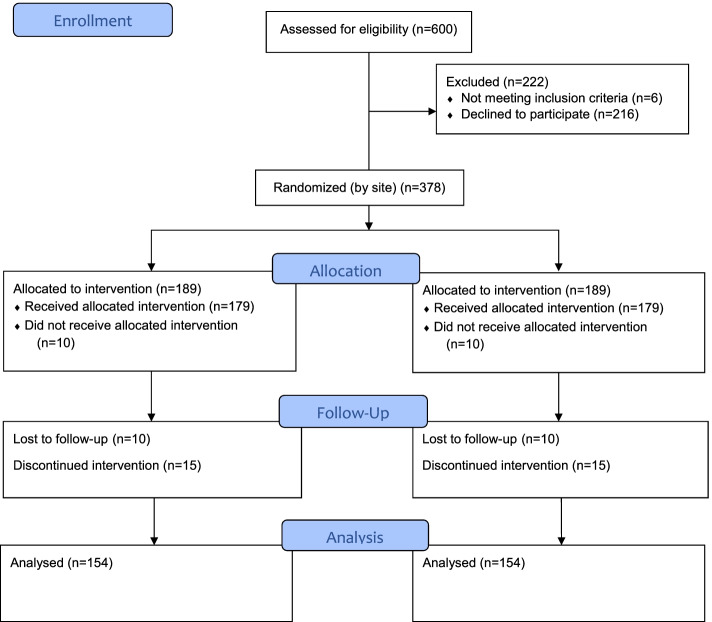
CONSORT 2010 Flow Diagram (Anticipated Numbers)

We expect that each treatment site will enroll on average about 16 participants, yielding a total of 378 participants (see Fig. 2). For all power analyses conducted, based on previous experience, we assumed 20% attrition by 12 months. We further assumed four assessment points (baseline, 6-, 12-, 18- and, for 75% of the sample, 24-month), an alpha level of 0.05, a two-tailed test, with a cross-time correlation of 0.5. We will minimize treatment dropout by reaching out to participants whenever they miss treatment sessions (through the site RA) and by providing makeup sessions (through the site clinicians) that allow those who have missed up to eight sessions to reengage in the treatment without missing content. We will minimize attrition from the research procedures by identifying multiple contact persons when enrolling each participant and by using multiple forms of outreach when a participant is not available for scheduling an assessment or misses a scheduled assessment. Based on our prior experience conducting clinical trials evaluating CET and HOPES (reviewed above), combined with our methods for minimizing attrition, we also believe it is realistic to estimate that the attrition rate for the 1-year durability follow-up will not exceed 20%. However, to be conservative we have estimated our power to detect significant differences between the groups based on a 25% attrition rate.

To estimate effect sizes, we compared effect sizes of CET and SST/HOPES from our own data and the SST literature using randomized controlled designs in schizophrenia, bearing in mind the diversity of design and treatment length issues (see Table 1). Given that CET is a composite intervention that includes both cognition, social cognition and social skills interventions, the effect sizes are not surprisingly higher (from .7 after 1 year to > 1.0 after 2 years); SST effect sizes are mostly under .4 in meta-analyses of SST of lengths from 3 weeks to 2 years (and in the HOPES trial did not consistently lessen for one or even 2 years after active treatment). Based on the available studies outlined in the table, we estimated effect sizes of 0.34 (for ICC due to clustering at site level = .01) and 0.37 (for ICC = .02), which justifies our proposed full sample size. With 4 assessments for the full and 5 assessments for three-fourths of the sample, our durability assessment is only somewhat less powerful, with medium effect sizes (from 0.41 to 0.49).

*Accounting for clustering and correlated data.* There are multiple forms of clustering due to participants being nested in sites and repeated observations being nested in participants. Therapeutic groups within a site can also be considered as another level of clustering, but because there are no more than 3 therapeutic groups at any site and they are expected to be led by the same clinicians at the site, we expect little between-therapeutic group heterogeneity within sites and so this level of clustering is unlikely to affect estimates. Nonetheless, we conducted two sets of power analyses, with and without considering therapeutic groups as another level of clustering.

We first only consider clustering effects due to sites and repeated measurement and handle clustering effects as follows: for the correlated nature of data due to repeated measurement, we assumed within-person cross-time correlation to be 0.50 and incorporated this correlation into both analytic modeling procedures (mixed-effects model with random effects) and power calculations. To account for site level clustering effects, we adjusted for sample sizes of 378 individuals by assuming a range of ICC values: 0.01, 0.02 and 0.05. We note the literature reports that in public health and medicine, ICCs in group- or cluster-randomized trials are often small, usually ranging from 0.01 - 0.05 (https://researchmethodsresources.nih.gov/FAQs.aspx). Some statisticians have stated that values of ICCs are usually between 0.01 and 0.02 in cluster randomized clinical studies [78, 79]. Given the multi-determined nature of our health-related outcomes, we believe that the effects due to clustering by site in our study will be small, and thus we adjusted our sample size and power calculations based on two scenarios: assuming an ICC of 0.01 and an ICC of 0.02. After adjustment, our original sample size of 378 reduced to an effective sample size (ESS) of 318 (for an ICC of 0.01) and 274 (for an ICC of 0.02), respectively. Second, for the 3-level clustering effects adjustment, we took account of the 51 therapeutic groups (approximating) as another level of clustering and assumed an ICC due to therapeutic group of 0.01, and adjusted sample sizes by both site and therapeutic group down to 302 and 252, respectively.

#### Power for aim 1

Power analyses for Aim 1 were conducted using Diggle et al. [80] and Lu et al.’s [81] approaches implemented in the LongPower package [82] in R statistical software (version 3.52). Power calculations were conducted to determine minimum detectable differences between arms (CET vs. SST) in changes over time on the primary outcomes. The outcomes for primary hypotheses in Aim 1 are continuous. To detect significantly more improvement for participants in CET than SST with 80% power in the 2-level analysis, the adjusted sample size of N of 318 (with 20% attrition by 12 months) will detect a minimum effect size of 0.34, and the adjusted N of 274 will detect a minimum effect size of 0.37, respectively. Both detectable effect sizes are between small and medium according to Cohen’s d metric for effect size (0.2 = small, 0.5 = medium and 0.8 = large). For the 3-level analysis that includes adjustment for clustering at the therapeutic group level and the adjusted sample sizes of 302 and 252 (everything else is the same), the minimum effect sizes that can be detected are 0.35 and 0.38, respectively.

The full sample of 378 at baseline (thus ICC adjusted sample sizes of 318 and 274) will have 4 assessments (baseline, 6-, 12 and 18 months), but we will also collect data for 284 participants (75% of the original sample of 378) for one extra time point (baseline, 6-, 12-, 18 and 24 months) (thus an ICC adjusted effective sample size for *N* = 284 participants becomes 250—for an ICC of 0.01—and 222—for an ICC of 0.02—respectively, and so the detectable effect sizes with *N* = 250 and 222 with 20% attrition are 0.38 and 0.40, respectively. For the analysis with 3-level clustering (both site and therapeutic group, as well as participants over time), the adjusted sample sizes become *N* = 240 and 208 and the detectable effect sizes with 20% attrition are 0.39 and 0.42, respectively.

### Measures

All interview ratings and cognitive testing will be conducted by trained raters and administered over the web by a project research assistant at the project’s central site, with an on-site RA managing arrangements so that the interviewer is blind to treatment assignment. Participants will be assessed at Baseline, 6 months into the program, 12 months (at completion of the program), 18 months (6 months after program completion), as well as 24 months (12 months after program completion for at least 75% of participants—those whose groups have completed in sufficient time). All data are recorded at the time of assessment in BIDMC’s secure and HIPAA-compliant REDCap system and are subsequently checked for completeness by the project research coordinator.

The outcomes we have chosen include the primary and secondary outcomes identified in prior research on CET and SST/HOPES and represented in our hypothesized causal model (see Table 2). The measures are easy to administer at the community level, endorsed by the stakeholders, relatively brief (total < 2 hours for the overall assessment), and include engaging qualitative questioning as well as fixed choice questions. Since all assessment procedures were adapted for videoconferencing due to the pandemic, several measures from the Penn CNB that can be administered remotely replace corresponding measures in the NIH Toolbox that have not been adapted for remote delivery.

**Table 2:** Outcome Measures and Causal Role

Category	Role
**Outcome Measures**	
*PANSS-6*	Negative symptoms
Neurocognition: NIH Toolbox and Penn CNB	Neurocognition
Social cognition: MSCEIT: Managing Emotions	Social cognition
Social Skills Performance Assessment (SSPA)	Social functioning
Community functioning (SAS-II)	Community functioning
Heinrichs Quality of Life Scale	Quality of Life
**Baseline-only measures**	
Diagnosis: MINI	Eligibility
Estimated IQ (from NIH Toolbox)	Moderation

#### Primary outcome measures

*Community functioning* is measured with the Social Adjustment Scale (SAS-II), which has previously been validated as an interview-based measure (utilizing self-reports) of social and community functioning [83]. The SAS-II quantifies adjustment in different roles (e.g., work, school), social and leisure activities, intimate relationships, and overall adjustment. *Quality of life* is assessed with the Quality of Life Scale [84]. This 21-question interview-based, interviewer-rated measure assesses social functioning, role functioning, and motivation, as indicated by items such as sense of purpose, curiosity, and inner drive [85]. To measure symptom severity, we will use a short, validated version of the Positive and Negative Syndrome Scale (PANSS-6, 6 items) [86, 87].

#### Secondary outcome measures

*Neurocognition*. We use a combination of measures from the NIH Toolbox and the Penn CNB that can be administered remotely and that match the 7 cognitive domains in the MATRICS Consensus Cognitive Battery. From the NIH Toolbox: Auditory Verbal Learning Test (verbal memory and learning), List Sorting Working Memory (working memory), Picture Sequence Memory (visual episodic memory and learning), Picture Vocabulary (language), and Oral Reading Recognition (language). From the Penn CNB: Continuous Performance Test (attention/vigilance), Mouse Practice Task (speed of processing), Digit Symbol (speed of processing), Conditional Exclusion Test (executive functioning/reasoning and problem solving). *Social cognition*. We use the Managing Emotions subtest of the Meyer-Salovey-Caruso Emotional Intelligence Test (MSCEIT) which is sensitive to treatment-related change in social cognition [88] and social functioning in SZ [56]. Social skills will be assessed using the Social Skills Performance Assessment (SSPA), a role play test that is audio-recorded and then rated for different dimensions of social skills [89].

#### Process evaluation

We will conduct a process evaluation using mixed methods to assess both intervention delivery and participant experience in each intervention (see Table 3). Treatment delivery will be monitored with three types of data. (1) *Treatment fidelity*: Group sessions are recorded at each site throughout the study and recordings are uploaded to a REDCap database. The recordings are monitored by supervising clinicians (study Co-Investigators) and used for feedback and training; at least six sessions are reviewed during the first 3 months and at least four sessions are reviewed thereafter through the remainder of the twelve-month treatment period. Supervising clinicians will rate at least ten sessions per year for fidelity with respect to each CET and HOPES group, using standard fidelity scales for both treatments that have been adapted to a common 4-point metric [40–42, 45]. (2) *Treatment engagement*: Treating clinicians and site RAs at each site create careful records of all individual and group sessions delivered, patient attendance, and session highlights. These records also include homework completion (CET, HOPES) community trips (HOPES), and makeup sessions (CET, HOPES). The clinicians and site RAs record this information and the site RA enters it in a REDCap database. (3) *Treatment experience*: The supervising clinicians of CET and SST summarize their impression of treatment delivery at each site at 6 and 12 months in qualitative comments; these summaries are captured in a REDCap database.

**Table 3:** Selected Process Measures

Process measures	Data collected by
Treatment satisfaction questionnaire (self-rating) [90]	Patient self-rating
Treatment fidelity (checklist)	Supervising clinician
Treatment adherence (provider-based)	Site RA^a^
Simplified Service utilization rating form (SURF) [91]	Research assistant

^a^Site RAs will be trained to collect these data

Participant experience is monitored with both quantitative and qualitative data. (4) *Participant satisfaction*: Participants are sent invitations to complete the Client Satisfaction Questionnaire (CSQ-8), a brief eight item self-report survey, via their mobile device, tablet, or computer once per month, during each month that they are enrolled in treatment [90]. (5) *Participant assessment*: Two randomly selected participants are selected during month-2 of treatment delivery at each site for a qualitative interview using an open-ended interview designed to elicit information about the treatment experience. This process is repeated using a similar open-ended interview schedule for two randomly selected participants in the 11th month of their treatment. (6) *Service utilization*: Participants continue to participate in regular services throughout the intervention, excepting other SST-based or CR-based therapies. A modified version of the Service Use and Resource Form (SURF) pertaining to services received in the past month is completed by participants at baseline and follow-up assessments [91]. (Treatment interruptions due to hospitalization or other events are recorded as part of the treatment engagement record).

We will monitor treatment delivery throughout the project and the project leadership will contact site PIs or other stakeholders as necessary to understand the bases for any indications of problems with treatment quality or quantity. Supplementary training will be provided to clinicians at sites as needed to ensure treatment fidelity and the project leadership group-- including engagement coordinators and staff—will review a summary report based on process measures each quarter to ensure identification of processes in need of improvement. If necessary, we will refine our recruitment and retention strategies based on our quarterly review of patient engagement.

### Planned analyses

#### Analysis for aim 1

All analyses will be conducted on the full sample regardless of exposure to treatment (intent-to-treat). Our primary hypothesis in Aim 1 is that CET will be associated with greater improvements than SST in both the *primary outcome*: community functioning (SAS, QLS), and the *secondary outcomes* of neuro- and social cognition (BACS and MSCEIT) and social skills (SSPA). To test this hypothesis, we will fit generalized linear mixed-effects models (GLMM) with identity link functions and a normal distribution specification for both the primary outcome and the secondary outcomes (because both outcomes are measured with interval-level scales). Because patients are nested within site, and repeated observations are nested within participants, we will fit a 3-level mixed-effects model: treatment arm (CET vs SST), time (baseline, 6-, 12- and 18-month) and arm-by-time interactions will be specified as fixed effects. The primary hypothesis of interest will be examined by testing the significance of the difference from zero in the rate of improvement over 18 months (arm-by-time interaction effect). To consider possible variance inflation due to clustering at the site and patient level, site will be treated as a random effect, and the intercept and slope for time at patient level will be specified as random effects. Randomization of sites to implement the CET or SST intervention will be stratified by geographic proximity, such that pairs of sites closest to each other will be randomized to different treatment conditions, and by organizational type and demographic characteristics of the surrounding area. This stratification plan will minimize the impact of any potentially confounding effects on the findings related to the bases of stratification. Because use of stratification may induce correlation between treatment arms, as a recommended cautionary measure, we will adjust for this stratification by including it as a random effect [92].

Because the interventions will be delivered in a group format sequentially for both arms, therapeutic groups could be considered as another level of clustering to be adjusted. We therefore will also test for effects with a 3-level model that includes the 51 treatment groups as another level of clustering. If variability (random effects) at the therapeutic group level is not significant, or if there is no improvement in model fit, we will remove therapeutic group as another level of clustering and retain the 2-level model. Whether a 2- or 3-level model is the final one chosen, we will specify heterogeneity of group effects (treatment arms) in the model to account for any potential variation between the treatment groups.

#### Analysis for Aim2 (HTE or moderation effect)

We will explore differential effectiveness of the two interventions by baseline cognitive functioning and age. Prior research suggests that these factors may influence effectiveness of CET and SST in different ways, but the evidence is not strong, and no studies have been conducted of both treatments. Therefore, we consider these hypotheses exploratory rather than confirmatory in nature. Prior to modeling, two moderators, baseline cognitive impairment and age will be recoded into two dichotomous variables using a median split, respectively.

In this aim, we test heterogeneity of treatment effects (HTE) or moderation effects by fitting the interaction models and conducting sub-group analyses. To test HTE, we will use the same analytic models—GLMM for Aim 1—but we will include 3-way interaction terms (arm*time*group) in the model. A significant 3-way interaction effect will indicate the existence of the heterogeneity of treatment effects between subgroups. Following this step, we will conduct simple effect analysis to estimate treatment effect differences (a difference in slopes by time effect between arms) within each subgroup.

Conceptually, the secondary outcomes of neurocognition and social cognition can be viewed as an intermediated outcome (mediator, M). The intervention (X) may affect the primary outcomes (Y) indirectly through a pathway of the mediator (M) as illustrated in Fig. 1. To test the mediated effect (or mechanism of change), we will conduct causal mediation analysis within the counterfactual framework [93, 94]. The moderation hypothesis in Aim 2 may also apply to the mediation effect. That is, the magnitude of the mediated treatment effect may also vary depending on age category (young vs. old) and pretreatment cognitive functioning (high vs. low), thus we will also conduct moderated causal mediation analysis by treating age and baseline cognitive function as moderators (MO).

Specifically, we will compute change scores (amount of improvement) from baseline to 18 months (for all subjects), and from baseline to 24 months (for 3/4 of subjects) for both primary outcomes and mediators for mediation analysis. For primary (or marginal) mediation analyses, we will estimate total effect of the treatment (TE), which can be decomposed into direct treatment effect (called natural direct effect, NDE) and indirect (or mediated) effect (called natural indirect effect, NIE); that is, TE = NDE + NIE with two-way decomposition. Bootstrap methods will be used to estimate standard errors and confidence intervals for mediation effects. A major advantage of the causal mediation method is that it allows testing and controlling for potential confounding effects due to non-random assignments of the mediators. With this approach, the percentage mediated effect can also be calculated. To test mediated effects potentially moderated by baseline cognitive function (high vs. low) and age category (young vs. old), moderated causal mediation analysis will be conducted to examine whether mediation patterns are the same for subgroups defined by moderators. Magnitude and percent of mediated effect between the subgroups (conditional causal mediation effect) will be compared.

Variation between our patients and across our treatment delivery sites in patient engagement may also influence the effectiveness of both interventions. We expect that greater treatment dose (sessions attended) will multiply gains in treatment impact on community functioning and quality of life for both CET and SST, thus moderating the impact of treatment along both primary causal pathways, but not changing predictions of the relative effectiveness of CET compared to SST.

As our HTE analyses are not based on testing explanatory hypotheses, we will report the corresponding *p*-values for hypothesis testing for these subgroup analyses. However, rather than relying on statistical significance testing alone, we will evaluate HTE using a combination of statistical significance and clinically meaningful difference with emphasis on the magnitude of treatment effects with standard errors (clinically meaningful effect sizes) as outlined in PCORI Methodology Standards [95] and recommended by statisticians [96].

#### Adjusting for multiple subgroup comparisons

Subgroup analysis for treatment effects (i.e., estimate of the treatment effect for each subgroup) is a common approach for HTE analysis, but it is susceptible to the issue of multiple post hoc analyses. For the analyses of primary and secondary hypotheses, it is necessary to control for possible Type I error due to multiple tests. However, our moderation analyses will test exploratory hypotheses, and thus we will report the results of statistical tests including both corrected and uncorrected probability levels. To this end, we will adjust the *p*-value to account for multiple tests for each subgroup analysis using the false discovery rate method [97] implemented in SAS software [98]. Subgroup analysis may also increase the likelihood of Type II error due to small sample sizes. Therefore, we will not rely on statistical tests and *p*-values alone to evaluate HTE; we will also examine estimated effect sizes (estimate divided by the standard deviation) and compute confidence intervals around these effect sizes.

#### Missing data modeling

We will use generalized linear mixed modeling (GLMM) to test all hypotheses. The GLMM uses all available data, thus allowing intermediate missing data and attrition in the outcome vector. GLMM yields valid inferences, assuming data are missing at random (MAR). However, it is possible that some outcome data may not be missing at random. Thus, it is important to test the MAR assumption. Because the distinction between MAR and not missing at random (NMAR) involves unobserved data, we cannot test whether the missing pattern is MAR or NMAR using the collected data [99]. We will use multiple empirical ways to check the MAR assumption, and to ensure stability of our estimates and inference. In addition, we will conduct sensitivity analyses using several NMAR models (e.g., selection model and pattern mixture model) [100] to see if the results from different NMAR models are consistent from our MAR model. At a minimum, we will check to see if the patterns and rates of attrition are different across intervention arms; if we find this to be true, we will use a pattern mixture model to refit our models and to evaluate potential biases associated with missing data.

Throughout, we used the SPIRIT reporting guidelines [101].

## Stakeholder engagement

**Fig. 3:**
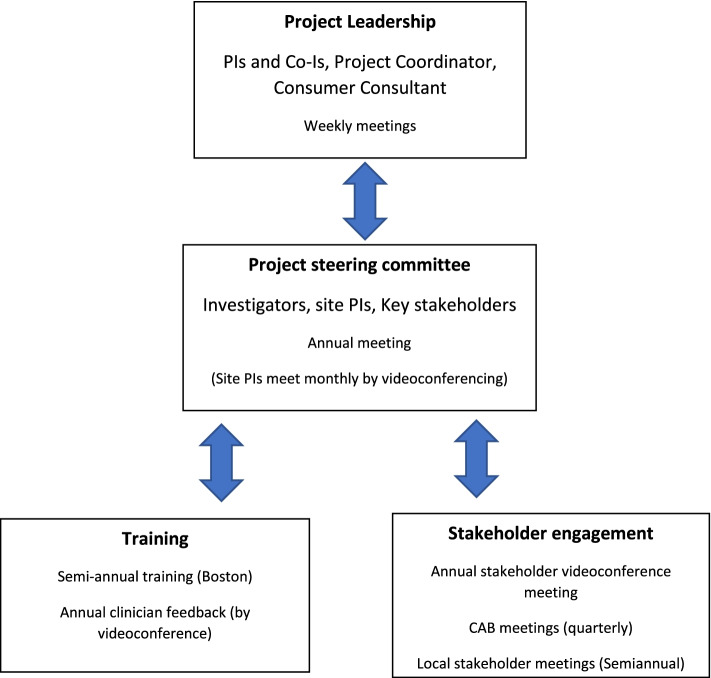
Project Leadership and Stakeholder Structure

The project leadership structure includes four groups: The Project Leadership Group, the combined Project Steering Committee, the Stakeholder Committee, and the Training and Implementation Team (CET and SST) (see Fig. 3). Local stakeholder representation is provided by the preexisting Consumer Advisory Board (CAB) at the Massachusetts Mental Health Center (MMHC)—where the study is headquartered within the BIDMC system—and by local stakeholder groups at other sites.

The Leadership Group is responsible for general project oversight, project planning, and management of implementation, training, treatment, engagement, and assessment, as well as planning study-related publications. Throughout the study, weekly meetings of the co-PIs and the entire co-investigator group and monthly meetings of the site PI group ensure coordination of all project activities and timely response to any challenges. Our lead consumer consultant will provide guidance as a cultural broker to ensure effective communication with participants and to maintain an appropriate balance of power throughout the project.

The Project Steering Committee provides overall project guidance in a collaborative structure that maximizes shared learning and site-specific lessons. This Committee’s annual meeting brings together the Project Leadership Group, the site PIs, and the Stakeholder Committee representatives to ensure multiple perspectives on project activities and critical engagement about project challenges. Many of the site PIs are very experienced researchers and all are experts in service design and delivery, while members of our Stakeholder Committee represent a wealth of experience in different service delivery systems and with different roles in the treatment system. At the project’s conclusion, a combined Stakeholder and Steering Committee meeting will present main research findings, provide breakout groups for discussion and interpretation, and plan publications.

The co-PIs will lead monthly virtual meetings of the site PIs to ensure flexible and timely response to site-specific challenges in implementation, management, and engagement as well as to maximize sharing of strategies and perspectives and communication of results.

A broad spectrum of stakeholders representing lived experience, advocacy, and organization and delivery of services will provide advice and feedback to guide the direction of the project so that the most effective interventions can be implemented widely following conclusion of the PCORI-sponsored project. Our Stakeholder Committee begins with 13 members representing 11 different locations and the perspectives of people with lived experience of mental illness (7 members), family members (2 members), and professionals (4 members). The Stakeholder Committee members will have two annual virtual meetings to review project progress, seek feedback about challenges to recruitment, data collection and other project activities, and discuss what we have learned from treatment delivery and preliminary analyses. Meetings will also address any site-level barriers to conducting the study.

The MMHC Consumer Advisory Board (CAB) represents a partnership between people with research, clinical and/or lived experience with SMI. The CAB at our project headquarters provides a unique local opportunity to elicit and receive feedback from individuals with lived experience who are not involved in the conduct of the study and are therefore able to offer suggestions and advice that may not occur to those who are project participants. These individuals already have deep experience in participatory clinical research. The MMHC CAB will review project reports at one meeting each quarter to provide regular and frequent input into recruitment, retention, intervention delivery, questions for qualitative interviews on treatment process, and suggestions for data analysis.

Patient representatives and advocates on the Steering Committee will hold semiannual meetings with the local stakeholder groups to review and inform treatment and research plans. We consider these meetings to be a vital part of our plan to manage our project in a way that reflects the values of social connection and peer support that are reflected in the interventions themselves. The local stakeholder groups represent the strategies developed at unique sites to engage stakeholders and so will provide diverse perspectives on project issues that in turn will result in a more holistic understanding of project progress.

The process of synthesizing stakeholder feedback will be dynamic, responsive, and person-centered. Stakeholder committee members will present reports about local stakeholder groups (including the CAB) at the biennial Stakeholder Steering Committee meetings, as well as at the annual project Steering Committee meeting. An ongoing working group of Stakeholder Committee members will periodically review process evaluation and assessment data and suggest improvements in procedures and directions for analyses. Collaboration and partnership among these groups is facilitated by leadership provided by our consumer consultant, Dr. Jon Delman, and a Stakeholder Leadership Team that also includes one of the two co-PIs and two co-investigators [102].

When stakeholder feedback suggests or the Steering Committee participants recommend changes, the leadership team will propose actions and then seek review by the stakeholder groups. After taking feedback into account, the leadership team will publicize and implement the resulting recommendations. Any indications of adverse events and any protocol modifications are reported to (and must be approved by) the BIDMC CCI (IRB) and trial conduct is also monitored by PCORI in monthly meetings and semi-annual reports.

All co-investigators are expected to be co-authors of major project papers and will serve as an approval board to grant authorship to study staff, site-level principal investigators, and stakeholder committee members upon request and when warranted by expert contributions to specific articles.

## Discussion

Since the advent of deinstitutionalization beginning in the 1960s, leaders in psychosocial rehabilitation have recognized the problems for community functioning caused by deficient social skills and have developed and refined training programs to overcome these deficits. The efficacy of multiple SST programs has now been established. It is only more recently, over the last three decades, that the cognitive impairments associated with SZ have become the focus of systematic remediation efforts [103]. The resulting body of research makes it clear that adding cognitive remediation to a psychosocial intervention improves functional outcomes more than providing psychosocial intervention alone [104] and that cognitive remediation programs added to psychosocial interventions have stronger effects on functional outcomes than “stand-alone” cognitive remediation interventions that only target cognitive functioning [32, 36, 37]. What has remained unknown has been whether these two different psychosocial treatments are equally effective on average for the improvement of community functioning and whether one or the other is advantageous for some affected individuals. As a result, clinicians have no empirical basis for deciding which treatment to recommend nor determining which individuals may benefit most from which treatment. Our PCORI-funded comparative effectiveness trial will achieve a head-to-head comparison of SST and CET and so will provide guidance to permit choice of intervention.

The resource shortages endemic in mental health services and exacerbated by the pandemic heighten the importance of this investigation [91]. Organizations that use the more comprehensive approach to psychosocial rehabilitation, which includes remediation of deficits in both neurocognition and social cognition, require additional training of personnel and cost more to deliver. It is imperative to determine whether the potential benefits justify the additional costs. In an era when facility with and interest in computer-based exercises varies with age and cognitive ability, the efficiency of resource allocation will also be improved by our test of whether cognitive remediation is more beneficial for younger persons and/or for those more cognitively impaired.

The experimental design of our research allows a well-powered, internally valid test of the two interventions as well as systematic exploration of several key hypothesized interactions between the interventions and participant characteristics. Our comprehensive assessment battery, with multiple assessments during and after the intervention experience, allows us to contrast the two interventions in terms of different dimensions of social, cognitive, and community functioning, as well as to evaluate mechanisms hypothesized to underlie the primary effects of each treatment.

Inclusion of community stakeholders is essential in community-based research to develop implementation strategies that reflect the specific opportunities and constraints in different settings as well as to build support for and publicize the value of the interventions. Our leadership structure creates a bidirectional process of exchange between research personnel and stakeholder groups that include persons with lived experience, advocates, and clinicians. By engaging stakeholders in a project-wide advisory committee as well as in site-specific groups, we will be able to maximize the input we receive and refine the guidelines we develop.

The pandemic created special challenges for delivery of socially engaging mental health services. We conducted a pilot study to adapt our treatments and methodology to these changed circumstances and will report the results in a separate article]. These adaptations do not change the treatment elements or research methods described in this article.

Overall, we believe that the proposed comparative effectiveness trial addresses a key knowledge gap for the field and a decisional dilemma facing clinicians seeking to improve community functioning in schizophrenia. Given that schizophrenia is one of the most disabling illnesses in all of medicine, the public health significance of the research is substantial.

## Data Availability

The datasets during and/or analysed during the current study as well as the full protocol and model consent form will be available from the corresponding author on reasonable request.

## Abbreviations

PCORI: Patient-Centered Outcomes Research Institute
CET: Cognitive Enhancement Therapy
HOPES: Helping Ourselves Pursue and Experience Success
SZ: Schizophrenia
SST: Social Skills Training
CR: Cognitive remediation
RCT: Randomized clinical trial
CLUES: Cognition for Learning and for Understanding Everyday Social Situations
MINI: Mini International Neuropsychiatric Interview
DSM-5: Diagnostic and Statistical Manual 5
BIDMC: Beth Israel Deaconess Medical Center
ICC: Intra-cluster correlation coefficient
HIPAA: Health Insurance Portability and Accountability Act
SAS-II: Social Adjustment Scale
PANSS-6: Positive and Negative Syndrome Scale
Penn CNB: Penn Computerized Neurocognitive Battery
MSCEIT: Meyer-Salovey-Caruso Emotional Intelligence Test
SSPA: Social Skills Performance Assessment
CSQ-8: Client Satisfaction Questionnaire
NIH: National Institutes of Health
SURF: Service Use and Resource Form
BACS: Brief Assessment of Cognition in Schizophrenia
GLMM: Generalized linear mixed-effects models
HTE: Heterogeneity of treatment effects
SAS: Statistical Analysis System
MAR: Missing at random
NMAR: Not missing at random
CAB: Consumer Advisory Board
MMHC: Massachusetts Mental Health Center
SPIRIT: Standard Protocol Items: Recommendations for Interventional Trials
SMI: Serious mental illness
