# Correction: Cognitive Enhancement Therapy vs social skills training in schizophrenia: a cluster randomized comparative effectiveness evaluation

**DOI:** 10.1186/s12888-022-04250-1

**Published:** 2022-09-16

**Authors:** Russell K. Schutt, Haiyi Xie, Kim T. Mueser, Matthew A. Killam, Jonathan Delman, Shaun M. Eack, Raquelle Mesholam-Gately, Sarah I. Pratt, Luis Sandoval, Meghan M. Santos, Laura R. Golden, Matcheri S. Keshavan

**Affiliations:** 1 Beth Israel Deaconess Medical Center, Harvard Medical School, Boston, USA; 2 University of Massachusetts Boston, Boston, USA; 3 Dartmouth College, Hanover, USA; 4 Boston University, Boston, USA; 5 University of Massachusetts Medical School, Worcester, USA; 6 University of Pittsburgh, Pittsburgh, USA; 7 Geisel School of Medicine at Dartmouth, Hanover, USA

**Correction: BMC Psychiatry 22, 583 (2022)**

**https://doi.org/10.1186/s12888-022-04149-x**

Following publication of the original article [1], the authors identified an error in the author name of Haiyi Xie.

The incorrect author name is: Haiyi Xi

The correct author name is: Haiyi Xie

The author group has been updated above and the original article [1] has been corrected.
